# Thermochromic nacre-inspired structural material

**DOI:** 10.1093/nsr/nwaf191

**Published:** 2025-05-15

**Authors:** Yucong Su, Yadong Yin

**Affiliations:** Department of Chemistry, University of California, Riverside, USA; Department of Chemistry, University of California, Riverside, USA

High-performance biomimetic structural materials with enhanced strength and toughness can be fabricated by mimicking the highly ordered ‘brick-and-mortar’ architecture of natural nacre and incorporating a mineral bridge reinforcement mechanism [1,2]. Nevertheless, as the demand for multifunctional applications in complex and dynamic settings increases, these structural materials must also integrate additional adaptive features. In environments prone to fire, conventional biomimetic structural materials lack active fire-warning and passive flame-retardant capabilities. Recent advances in thermochromic materials integrated with image-recognition systems have shown significant promise in early-stage fire-warning applications [3]. These systems leverage heat-triggered color transitions to produce real-time visual signals, enabling rapid fire detection and response.

**Figure 1.**
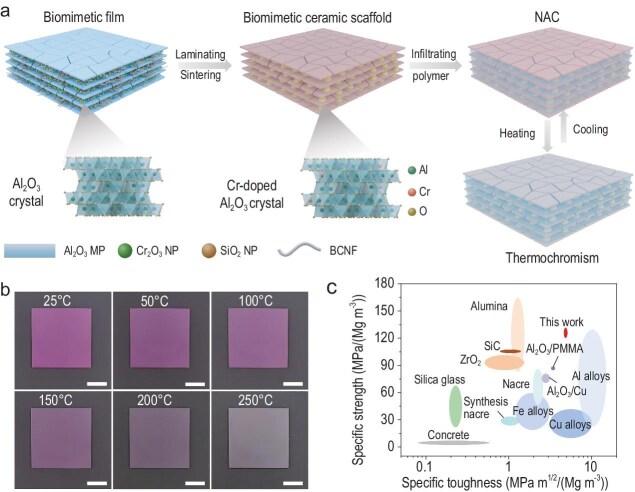
(a) Schematic illustration of multiscale structure design and fabrication of the biomimetic bulk composite. (b) Photographs showing the thermochromic change of the NAC from 25°C to 250°C. Scale bars, 1 cm. (c) Ashby diagram of specific strength and specific toughness for NACs compared with a series of engineering materials. Reproduced from Ref. [4] with permission.

In a recent study [4], Yu *et al*. developed a novel strategy that integrates atomic-level doping with a biomimetic structural design to fabricate nacre-inspired alumina-cyanate resin composites (NACs) exhibiting mechanical robustness, thermochromic responsiveness, and intrinsic flame-retardant functionality. The Cr-doped alumina micro-platelets (Al_2_O_3_ MPs) were synthesized via a controllable solid-solution reaction and utilized as the ‘bricks’ in the biomimetic nacre structure (Fig. 1a). This doping process endows the composite with thermochromic behavior derived from ligand field transitions within the d orbitals of Cr^3+^. As shown in Fig. 1b, at ambient conditions, Cr-doped Al_2_O_3_ appears red due to crystal field splitting. Upon exposure to high temperatures, lattice expansion reduces the constraint of the O^2–^ anions on the Cr^3+^ valence orbitals, shifting the absorption spectrum and causing a reversible color transition to green. Meanwhile, this atomic doping strategy contributes to solid-solution strengthening, suppressing transcrystalline fracture and significantly improving the composite's mechanical performance, achieving a light weight (∼2.3 g cm^−3^), high flexural strength (∼290.1 MPa), and high fracture toughness (∼11.1 MPa m^1/2^) (Fig. 1c).

Due to the thermochromic properties of the NAC, it can function as an early fire warning material that demonstrates sensitive responses prior to the emergence of flames. The integration of image recognition technology based on a machine learning K-means model allows for a rapid thermal response and alarm activation within 9 seconds at 250°C, validating its effectiveness for early-stage fire warning. Additionally, the anisotropic layered structure enhances out-of-plane thermal conductivity after resin infiltration, accelerating heat dissipation and improving thermochromic responsiveness. At high temperatures, the layered microstructure also acts as an oxygen diffusion barrier, achieving an oxygen-limiting index of 50%, thereby providing passive flame-retardant and fire-protective properties.

This interesting work demonstrates the successful integration of thermochromic signaling and flame resistance within a nacre-inspired composite framework. The resulting material establishes an active-passive synergistic fire protection strategy, offering structural integrity, early-stage thermal alarm capability, and sustained flame retardancy. This multifunctional system represents a significant advance in the design of smart structural materials for high-risk applications, particularly in environments where both mechanical resilience and intelligent fire response are critical.
